# Pan‐cancer analysis and oncogenic implications of *MGAM* and *MGAM2*: Toward precision oncology and drug repurposing in colorectal cancer

**DOI:** 10.1002/ccs3.70042

**Published:** 2025-08-27

**Authors:** Rawaa Chasib Mezher, Hanieh Azari, Reza Khayami, Hamid Fiuji, Farzaneh Alizadeh, Ladan Goshayeshi, Majid Rajabian, Ibrahim Saeed Gataa, Seyed Mahdi Hassanian, Amir Avan

**Affiliations:** ^1^ Metabolic Syndrome Research Center Mashhad University of Medical Sciences Mashhad Iran; ^2^ Department of Medical Biochemistry Faculty of Medicine Mashhad University of Medical Sciences Mashhad Iran; ^3^ Medical Genetics Research Center Mashhad University of Medical Sciences Mashhad Iran; ^4^ Department of Medical Oncology Cancer Center Amsterdam Amsterdam U.M.C. VU University Medical Center (VUMC) Amsterdam the Netherlands; ^5^ Department of Gastroenterology and Hepatology Faculty of Medicine Mashhad University of Medical Sciences Mashhad Iran; ^6^ Surgical Oncology Research Center Mashhad University of Medical Sciences Mashhad Iran; ^7^ Department of Biology Payame Noor University Tehran Iran; ^8^ College of Medicine University of Warith Al‐Anbiyaa Karbala Iraq

**Keywords:** acarbose; diagnostic, colorectal cancer, drug repurposing, *MGAM*, *MGAM2*, pan‐cancer, prognostic, RNA‐seq, voglibose

## Abstract

Cancer remains a major global health challenge, with high prevalence and mortality rates emphasizing the urgent need for innovative treatment strategies. Although precision oncology offers tailored therapies based on genetic profiles, the clinical translation of genomic insights has been slow. Drug repurposing, using existing FDA‐approved drugs for new indications, presents a cost‐effective and time‐efficient alternative. This study investigates *MGAM* as a potential direct target of alpha‐glucosidase inhibitors in colorectal cancer (CRC), explores its biomarker potential, and evaluates gene expression patterns across diverse cancers. Using RNA‐Seq data from Recount3, Firebrowse, and gene set co‐expression analysis databases, we analyzed the differential expression of *MGAM* and its paralog *MGAM2* across 33 cancer types. We examined mutation profiles, methylation status, survival impact, immune cell infiltration, and drug‐mRNA interactions. Validation was performed via real‐time PCR and whole‐exome sequencing (WES) in CRC patients. *MGAM* and *MGAM2* showed differential expression across multiple cancers, with *MGAM2* upregulated and *MGAM* downregulated in gastrointestinal tumors. Both genes were linked to key cancer‐related pathways, including metabolism, apoptosis, cell cycle regulation, and epithelial‐mesenchymal transition. *MGAM* exhibited frequent mutations and aberrant methylation in several cancers. Their expression correlated with immune cell infiltration and drug sensitivity, highlighting potential for therapy planning. Diagnostic modeling showed over 80% accuracy. In CRC patients, *MGAM* downregulation was confirmed in 64 samples, and WES revealed a novel *MGAM* mutation (rs2960746). These findings underscore *MGAM* and *MGAM2* as promising biomarkers and therapeutic targets, supporting their relevance in advancing personalized oncology.

## Graphical abstract

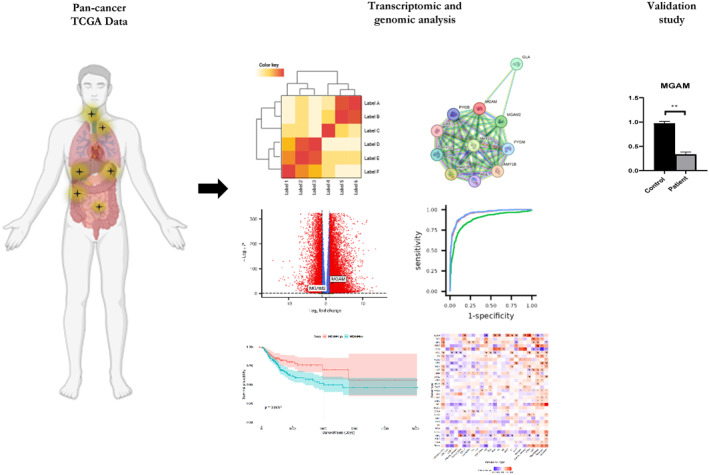

## 1 INTRODUCTION

Cancer is one of the leading causes of death worldwide and poses a significant challenge to global health, carrying a substantial social and economic burden.^1^ According to estimates from the International Agency for Research on Cancer (IARC), nearly 20 million new cases of cancer were diagnosed in 2022, with the number of deaths attributed to cancer being estimated to be 9.7 million.^2^ Despite significant advancements in surgical, radiation, and immunological‐based treatments that have enhanced the effectiveness of cancer therapies, the intricate and varied nature of the disease continues to pose significant challenges. Hence, there is an urgent need to address the complexities inherent in treating different types of cancer.^3^ Precision oncology utilizes specific characteristics of cancer patients, such as their genetic profiles, to recommend new personalized treatment regimens. However, despite extensive pharmaceutical research efforts to develop new drugs targeting these genes, clinical implementation of these discoveries has lagged behind expectations.^4^

Drug repurposing, also referred to as drug repositioning, presents an attractive and promising approach to treatment. Its advantages include cost‐effectiveness, expedited development timelines, and enhanced safety profiles, which stem from the comprehensive understanding of the drug's pharmacokinetics and pharmacodynamics. This method entails discovering novel therapeutic applications for medications that have already received regulatory approval.^3^, ^5^, ^6^ Drug repurposing employs several methodologies, including disease‐centric, target‐centric, and drug‐centric approaches, each with its unique focus and benefits.^7^ The **disease‐centric** approach initiates with the selection of a specific disease. Researchers then sift through approved drugs used for other ailments to determine if any could offer beneficial effects against the chosen disease.^8^ This strategy capitalizes on the extensive knowledge base surrounding the disease, seeking novel therapeutic applications for drugs already recognized for their efficacy in other contexts. The **target‐centric** approach, conversely, starts with a particular biological target, such as a protein or gene. Researchers pinpoint drugs that engage with this target and assess their capacity to address various diseases. This methodology is deeply grounded in the molecular mechanisms underlying disease pathology, aiming to uncover potential therapeutic agents based on a clear understanding of disease biology.^9^ Lastly, the **drug‐centric** approach selects an individual drug and explores its potential utility across a range of diseases. The goal here is to unlock the full therapeutic potential of a single drug by identifying additional conditions it may effectively treat, thereby maximizing its clinical impact.^3^, ^10^ Each of these methodologies comes with its own set of advantages and challenges. The synergy between them, however, can lead to the development of more effective and innovative treatments for diseases. By harnessing the strengths of each approach, researchers can enhance drug repurposing efforts, ultimately leading to improved patient outcomes.^7^, ^10^

The development of high‐throughput omics analysis techniques and the utilization of tools for big data analysis, facilitated by the Cancer Genome Atlas (TCGA), have progressively outlined the landscape of changes in signaling pathways.^11^ With the increasing application of multi‐omics approaches, the cancer research community can explore new opportunities for drug repurposing to target oncogenic stimuli in cancer patients.^12^ The likelihood of a drug being repurposed for cancer treatment increases significantly when its initially approved targets align with cancer‐related targets. This may arise when medications or therapies were initially designed and received approval for indications other than cancer, yet they interact with biological pathways or molecules that play a role in cancer development or progression. The rationale behind this potential effectiveness lies in the observation that numerous diseases, including cancer, often share common underlying mechanisms or molecular targets.^13^

Recent research has revealed that α‐glucosidase inhibitors such as acarbose, voglibose, and miglitol, commonly prescribed for diabetes management, exhibit antineoplastic properties.^14^, ^15^, ^16^ It was discovered that these inhibitors exert a beneficial effect on the risk of colorectal cancer (CRC) by influencing the pattern of bile acids in the stool and decreasing the levels of neutral sterols.^17^ Moreover, carbohydrate malabsorption has been suggested to potentially offer protection against colon cancer. A specific example of this protective effect comes from research involving the drug acarbose. The study demonstrated that acarbose could significantly lower the risk of developing CRC among diabetic patients. Importantly, the reduction in cancer risk appeared to be directly related to the dosage of acarbose administered, indicating a dose‐dependent relationship between the drug and its protective effects against CRC.^17^ On the other hand, cancer cells are recognized to have a higher demand for glucose, necessitating increased energy compared to normal cells.^18^ Alpha‐glucosidase inhibitors can effectively curtail the caloric intake of tumor cells by lowering blood glucose levels, ultimately leading to the destruction of tumor cells.^19^

The *MGAM* (maltase‐glucoamylase) gene, which encodes the enzyme maltase‐glucoamylase, is one of the alpha‐glycosidases found in the intestinal brush border membrane. This enzyme plays a crucial role in the final stage of carbohydrate digestion. The *MGAM* gene is located on chromosome 7q34. Because of its importance in carbohydrate metabolism, *MGAM* has been a major therapeutic target for treating type 2 diabetes and insulin resistance.^20^, ^21^, ^22^ *MGAM* binds to the epithelial cells of the brush border in the small intestine.^23^ On the other hand, genetic aberrations in chromosome 7 are commonly observed in a wide range of human diseases, including cancer.^24^

This research endeavors to explore the potential of *MGAM* as a therapeutic agent in cancer treatment through a multifaceted approach. Building upon the established effectiveness of alpha‐glucosidase inhibitors in CRC, as previously outlined in a preliminary literature review, we will first examine the expression of *MGAM* as a direct target of these drugs in CRC. Subsequently, we will expand our analysis to a broad spectrum of human cancers to identify patterns of *MGAM* expression, mutations, copy number variations (CNVs), methylation status, immune cell infiltration, and associated drugs across various malignancies. Our goal is to determine whether *MGAM* could serve as a pan‐cancer therapeutic target based on observed expression/mutation patterns across different cancer types. In addition to our current research, we seek to elucidate the molecular mechanisms that underlie *MGAM*'s function and its protein–protein interactions. We will also assess its potential as both a prognostic and diagnostic marker in cancers. This comprehensive approach will provide valuable insights into the therapeutic potential of alpha‐glucosidase inhibitors for cancer treatment.

The findings of this research will contribute significantly to our understanding of how genetic alterations affect drug efficacy across various cancer types. These insights will inform future clinical trials and guide the development of personalized treatment strategies for cancer patients.

## 2 MATERIALS AND METHODS

### 2.1 Data collection and identification of differentially expressed genes

To investigate *MGAM* expression in CRC, we utilized the TCGA‐COAD dataset retrieved from The Cancer Genome Atlas (TCGA) (http://cancergenome.nih.gov/). We focused on the white population to minimize heterogeneity and gain deeper insights by examining gene expression across different stages. After excluding cases with missing clinical data, we conducted gene expression analysis on a total of 214 patient samples and 17 healthy controls from the white population, as detailed in our previous work.^25^ We divided patients into three distinct subgroups based on tumor stage: one group combining stages I and II, another with stage III, and the last one with stage IV patients. Subsequently, we utilized the DESeq2 package to perform differential expression analysis within each subgroup separately. The criteria for significant differential expression were set at an adjusted *p*‐value <0.05 and a log fold change (|logFC|) ≥ 1.5.

In the next step to investigate the expression of *MGAM* in a pan‐cancer context, we used the recount3 project (RNA.recount.bio), which provided batch‐corrected data comprising 20,753 samples from TCGA and the Genotype‐Tissue Expression (GTEx) portal. This dataset included 11,094 normal and 9569 tumor samples. Using the edgeR software, we identified differentially expressed genes (DEGs) between the normal and tumor tissue samples. The significance threshold was set at |log2 (fold change)| ≥ 1 and adjusted *p*‐value ≤ 0.05.

The differential expression of candidate genes was further evaluated and confirmed across inter and intra‐cancer types using the Firebrowse^26^ and gene set cancer analysis (GSCA)^27^ online databases. These platforms employ the RSEM method, a software package designed to estimate gene and isoform expression levels from RNA‐Seq data.

### 2.2 Clinical samples

To maintain consistency and avoid redundancy, detailed demographic and clinical characteristics of the patient cohort have been previously published.^25^ Briefly, 64 patients diagnosed with non‐hereditary CRC were recruited, with RNA samples collected for qRT‐PCR validation of *MGAM* expression. Patient consent and ethical approval were obtained as described in the prior publication.^25^

### 2.3 Pathway enrichment analysis and PPI

The STRING database (https://string‐db.org)^28^ was utilized to identify all known and predicted physical interactions as well as protein–protein functional relationships of *MGAM*, with a minimum interaction score of 0.7 considered. In the following step, we utilized the GSCA database to examine the relationship between the expression levels of this particular gene and various regulatory elements within signaling pathways across all 33 cancer types.

### 2.4 Mutation profile assessment

The GSCA platform is a web‐based tool that integrates multi‐omics data from the TCGA database. Using this platform, we analyzed the profile of *MGAM* mutations, as well as the relationship between *MGAM* mRNA expression, CNVs, and single‐nucleotide variants (SNVs) across different tumor types. Additionally, we investigated the association between *MGAM* alterations, gene expression, and patient survival in all tumor samples.

### 2.5 Immune cell infiltration analysis

We then conducted an analysis using the GSCA database to examine the correlation between *MGAM* expression and the infiltration of 24 distinct types of immune cells. The immune cell abundance identity tool within this database provides estimates for the abundance of these immune cells, encompassing 18 subtypes of T cells and six other immune cell types (B cells, NK cells, monocytes, macrophages, neutrophils, and dendritic cells) across various cancer types.

### 2.6 Methylation analysis

The GSCA's module on differential methylation offers insights into the methylation status in cancer patients versus normal samples. The GSCA database analyzed the correlation between mRNA expression levels, *MGAM* methylation levels, and their impact on survival across various types of tumors.

### 2.7 Evaluating the prognostic significance of *MGAM* across different cancer types

Survival analysis was conducted on TCGA datasets utilizing the survival package in R. The study evaluated overall survival (OS) in various cancer types.

### 2.8 ROC curve analysis

The diagnostic capabilities of *MGAM*, both individually and in conjunction with *MGAM2*, the paralog of *MGAM*,^21^ were assessed using receiver operating characteristic (ROC) curve analysis. This evaluation emphasized critical performance indicators like sensitivity, specificity, and the area under the ROC curve. The analysis was performed utilizing the CombiROC package within the R programming environment.

### 2.9 Drug‐mRNA interaction

We employed the GSCA online database to explore drug‐mRNA interactions. This tool amalgamates half‐maximal inhibitory concentration (IC50) data from 250 small‐molecule drugs across 860 cell lines, alongside gene expression profiles sourced from the Genomics of Drug Sensitivity in Cancer (GDSC) initiative. It further computes the relationship between mRNA gene expression and drug IC50 values.

Additionally, we examined drug‐mRNA interactions through the drug–gene interaction database (DGIdb). DGIdb is engineered to streamline the identification of potential therapeutic targets within disease‐associated genes, particularly those linked to cancer. It alleviates the challenge of manually sorting through vast amounts of literature, clinical trial data, and databases to discover drug‐gene interactions. By leveraging expert curation and text mining techniques, DGIdb gathers information from reputable sources such as DrugBank, PharmGKB, and ChEMBL. This enables the classification of genes as potential targets based on their involvement in various pathways, molecular functions (MF), and gene families.^29^

### 2.10 Invitro validation studies

#### 2.10.1 Real‐time PCR

The expression of *MGAM* was validated through quantitative PCR (qRT‐PCR) following the isolation of RNA from 64 CRC samples and their corresponding controls using the ParsTous total RNA extraction kit from Tehran, Iran as described previously.^25^ Subsequently, the purity and concentration of the total RNA were assessed using a NanoDrop 2000 spectrometer. The extracted RNA was then reverse‐transcribed into cDNA according to the manufacturer's instructions using the ParsTous cDNA synthesis kit. The expression level of the *MGAM* gene was quantified using real‐time polymerase chain reaction (RT‐qPCR) with SYBR green master mix from ParsTous, Iran, and the ABI‐PRISM StepOne device. Graphical analysis and visualization were conducted using GraphPad Prism 10.0 software. *GAPDH* was utilized as an internal reference for RT‐qPCR data analysis, and the 2^−ΔΔCt^ method was employed to standardize the expression levels of the target genes.

The primer pairs utilized in this study were as follows:

*MGAM*‐Forward: GGCTGCAAGAGGTAATGAGAGAT, *MGAM*‐Reverse: CTGGGGCTGTTGATTTCAGTG, *GAPDH*‐Forward: ATCAGCAATGCCTCCTGCAC, and *GAPDH*‐Reverse: TGGTCATGAGTCCTTCCACG.

### 2.11 Whole exome sequencing (WES) analysis

Somatic mutations in the *MGAM* gene were investigated through an analysis of the TCGA database using the mutation annotation format (MAF). In the subsequent phase of the study, we also aimed to investigate and confirm *MGAM*‐related mutations in CRC patients. DNA extraction was carried out utilizing the ParsTous isolation kit from Tehran, Iran. This extraction was performed on blood samples obtained from 15 CRC patients following the manufacturer's protocol as described completely in our previous work.^25^ All identified variants underwent rigorous internal quality control and quality matrix assessment. Variants with a minor allelic frequency (MAF) equal to or exceeding 0.1% for heterozygous variants or 1% for homozygous variants were excluded based on data from the iranome and genome aggregation database (gnomAD). Finally, a suite of prediction tools, including sorting intolerant from tolerant (SIFT),^30^ polymorphism phenotyping version 2 (PolyPhen2),^31^ Varsome,^32^ and COSMIC,^33^ were employed to forecast the potential impact of non‐synonymous and synonymous (amino acid) alterations.

### 2.12 Statistical analysis

To assess the efficacy of the treatment, a range of statistical methods were utilized, including ROC curve analysis, Kaplan–Meier survival analysis, independent sample *t*‐tests, and χ2 tests. These analyses were conducted using R version 4.3.1, with statistical significance determined by a *p*‐value less than 0.05.

## 3 RESULTS

### 3.1 MGAM expression exhibits a stage‐specific marked decrease in CRC

**FIGURE 1:**
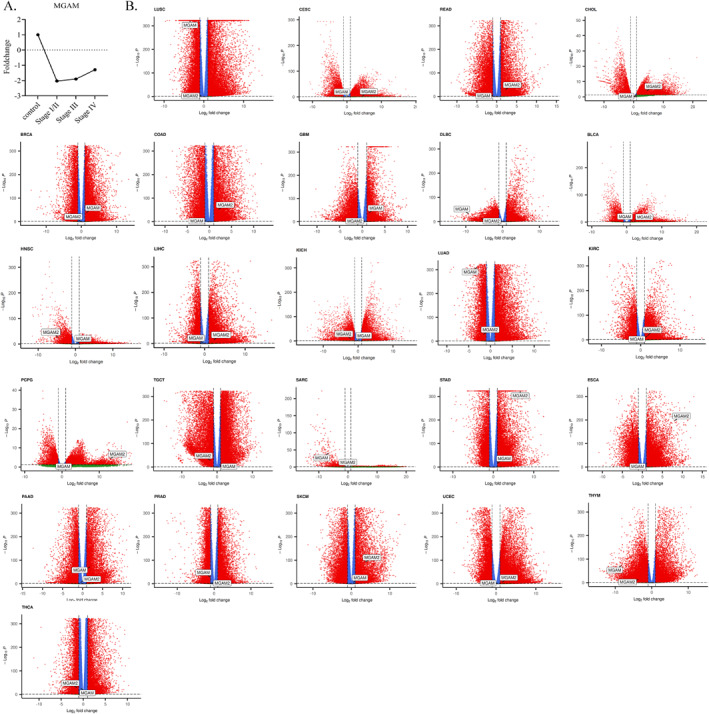
(A) Trend plot demonstrates the decrease in *MGAM* expression across stages using GraphPad Prism. (B) Differential expression of *MGAM* and *MGAM2* across several cancers based on data from the cancer genome atlas and GTEX, utilizing R software.

Analysis of the *MGAM* gene expression across different stages of CRC revealed a notable decline in expression levels. Specifically, *MGAM* expression decreased by 2.06‐fold in stages I and II, 1.9‐fold in stage III, and approximately 1.3‐fold in stage IV compared to control samples. Figure 1A shows the trend plot of MGAM expression across different stages.

### 3.2 Pan‐cancer analysis revealed differential expression of MGAM and MGAM2 across different GI cancers

We explored the differential expression of *MGAM* across various cancers, employing R software and online databases. Pan‐cancer analysis of RNA‐seq data highlighted significant differences in *MGAM* expression in urothelial bladder carcinoma (BLCA), lymphoid neoplasm diffuse large B‐cell lymphoma (DLBC), kidney renal clear cell carcinoma (KIRC), lung adenocarcinoma (LUAD), lung squamous cell carcinoma (LUSC), pancreatic adenocarcinoma (PAAD), sarcoma (SARC), and thymoma (THYM).

Additionally, the investigation extended to *MGAM*'s paralog, *MGAM2*, revealing its elevated expression in conditions such as carcinoma and endocervical adenocarcinoma (CESC), cholangiocarcinoma (CHOL), colon adenocarcinoma (COAD), esophageal squamous cell carcinoma (ESCA), KIRC, liver hepatocellular carcinoma (LIHC), pheochromocytoma and paraganglioma (PCPG), rectum adenocarcinoma (READ), skin cutaneous melanoma (SKCM), stomach adenocarcinoma (STAD), and downregulated expression in DLBC, head and neck squamous cell carcinoma (HNSC), kidney chromophobe (KICH), LUAD, testicular germ cell tumors (TGCT), and THYM (Figure 1B).

As previously mentioned, our RNA‐seq analysis is based on the data from TCGA tumors versus GTEX normal tissues. In this study, we also investigated the expression of these genes through the FireBrowse online database, which used TCGA tumors and matched normal tissue. Based on the results from these databases, a decrease in *MGAM* expression was also identified, notably in gastrointestinal cancers (GI) such as COAD, READ, CHOL, ESCA, LIHC, and STAD (Supporting Information S1: File 1). Notably, a strong relationship was observed between *MGAM* expression and subtypes of BRCA, KIRC, STAD, and HNSC, and *MGAM2* expression and subtypes of KIRC, BRCA, and BLCA (Supporting Information S1: File 2).

### 3.3 MGAM contributes to metabolic and cancer‐related pathways

**FIGURE 2:**
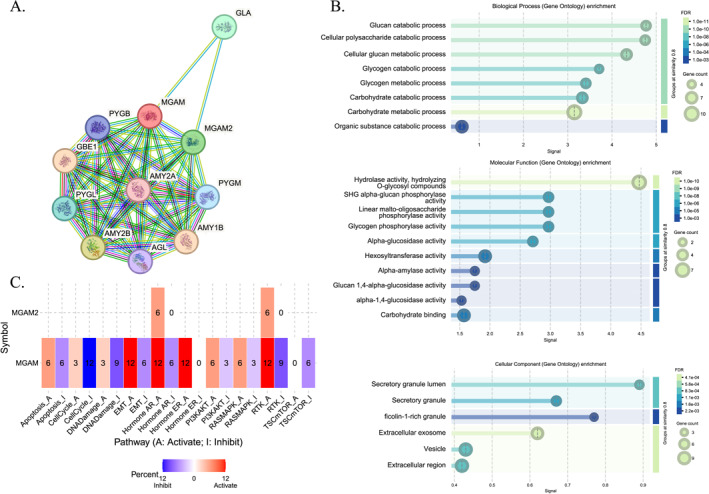
(A) Protein–protein interaction of *MGAM* utilizing the STRING database. (B) GO‐pathways in terms of biological process, molecular function, and cellular component. (C) Cancer‐related pathways regulated by *MGAM* and *MGAM2* across different cancers; the number in each box indicates the percentage of cancers associated with each pathway.

The STRING database has elucidated the protein–protein interaction (PPI) network of the *MGAM* gene, as shown in Figure 2A. Through gene ontology analysis, *MGAM* is identified as participating in numerous biological processes within metabolic pathways. It plays a significant role in the carbohydrate catabolic and metabolic process, the glucan catabolic process, and the cellular polysaccharide catabolic process, specifically in terms of biological function. Beyond these specific roles, *MGAM* demonstrated a range of MF in cooperation with *MGAM2*. These include glucan 1,4‐alpha‐glucosidase activity, alpha‐glucosidase activity, and alpha‐1,4‐glucosidase activity. Furthermore, when considering cellular components (CC), *MGAM* is linked to various structures such as ficolin‐1‐rich granules, secretory granule lumens, secretory granules, and extracellular exosomes (Figure 2B).

Our comprehensive analysis using the GCSA database revealed intricate relationships between *MGAM* and its paralog with cancer‐related pathways across various tumor types. Notably, *MGAM* exhibited divergent impacts on cellular processes in different cancer contexts (Figure 2C). In BRCA cancer, it was linked to the inhibition of key pathways, including apoptosis, cell cycle, DNA damage, RTK, PI3K/AKT, and TSC/mTOR while concurrently stimulating epithelial–mesenchymal transition (EMT), hormone AR, hormone endoplasmic reticulum (ER), and RAS/MAPK pathways. Interestingly, *MGAM* displayed contrasting effects on EMT in STAD and ESCA cancers compared to LIHC, LUSC, and HNSC. Furthermore, our data indicated that *MGAM* was associated with increased DNA damage in TGCT cancer and its inhibition in BRCA, KIRC, and LUSC cancers. The gene also demonstrated pathway‐specific activations and inhibitions across different cancer types, including the activation of apoptosis in LGG and LIHC, its inhibition in KIRC, and varied impacts on the cell cycle in ESCA, KIRC, and KIRP.

### 3.4 The mutation profile assessment proposed MGAM as a highly mutated gene in cancer

**FIGURE 3:**
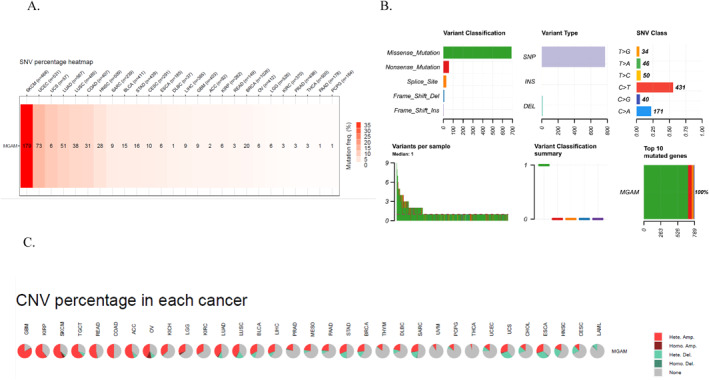
(A) The heatmap represents the *MGAM* single‐nucleotide variant percentage across different cancers using the gene set cancer analysis database. (B) Summary of *MGAM* variant classification. (C) Frequency of *MGAM* copy number variations across different cancers.

Further analysis of *MGAM* genetic changes revealed a high mutation rate in SKCM, UCEC, LUSC, LUAD, HNSC, COAD, BRCA, and STAD cancers (Figure 3A). Figure 3B illustrates the SNV classification in *MGAM*. The data revealed that missense mutations represent the most prevalent classification within *MGAM*. Notably, C > T transitions comprise the largest category of mutations observed. A significant correlation was observed between *MGAM* SNVs and OS and progression‐free survival (PFS) in PRAD cancer (Supporting Information S1: File 3). Figure 3C illustrates the frequency of CNVs across various cancers. Notably, a significant correlation was found between survival and CNV of the *MGAM* gene in UCEC cancer, considering OS, PFS, and disease‐specific survival (DSS) parameters (Supporting Information S1: File 4).

### 3.5 Immune cell infiltration analysis revealed the possible association of MGAM with immunity

The analysis of immune infiltration concerning the *MGAM* and *MGAM2* genes revealed an overall positive correlation with neutrophil and monocyte infiltration while exhibiting a negative correlation with CD4+, CD8+ T cells, and DC cells across various cancers such as KIRC, THCA, THYM, BRCA, LAML, PRAD, COAD, HNSC, LIHC, LUAD, STAD, and LGG. Particularly noteworthy is the strong correlation observed in LIHC, LGG, STAD, LUAD, SKCM, LAML, and HNSC between cell infiltration frequency and the expression of these genes (Figure 4A). The association of CNVs of *MGAM* with immune cells in different cancer types is illustrated in Figure 4B. As illustrated, amplification in *MGAM* seems to have more effect on immune cell abundance than deletion. Furthermore, SNVs of *MGAM* were identified to be correlated with higher Gamma delta, Th1, effector memory, nTreg, DC, and CD8‐T cells in cancers such as CESC, COAD, DLBC, LUAD, LUSC, SARC, SKCM, STAD, and UCEC, whereas they correlated with lower Th17, nTreg, macrophage, CD1, Th2, NKT, CD4‐Tcell, and Neutrophil in CESC, ESCA, HNSC, LIHC, LUAD, PRAD, SKCM, STAD, and UCEC (Figure 4C).

FIGURE 4(A) The correlation between *MGAM* expression and tumor infiltration. (B) Overall association of copy number variations in the *MGAM* across different cancers. (C) Overall association of SNVs in the *MGAM* across different cancers. All images were generated using the gene set cancer analysis database.

**Figure:**
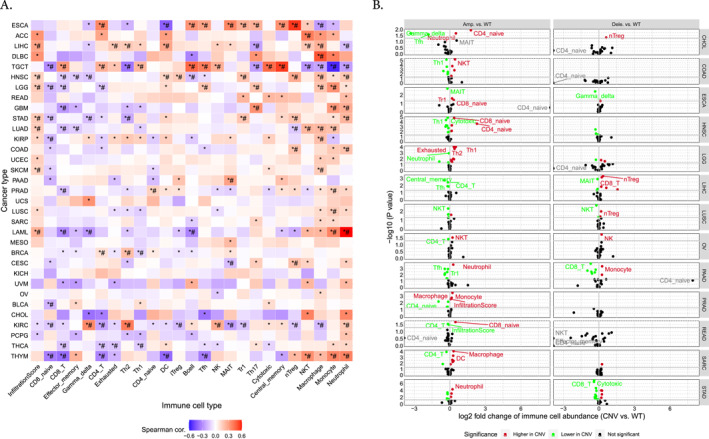

**Figure:**
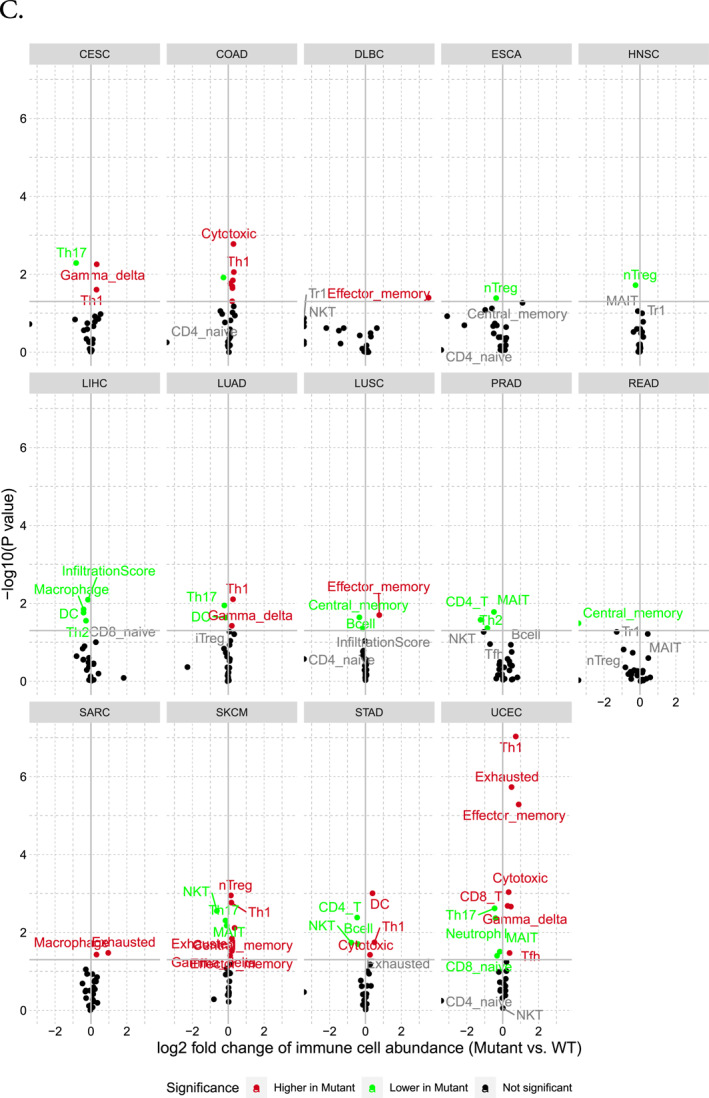

### 3.6 Abnormal methylation pattern in MGAM is associated with several cancers

**FIGURE 5:**
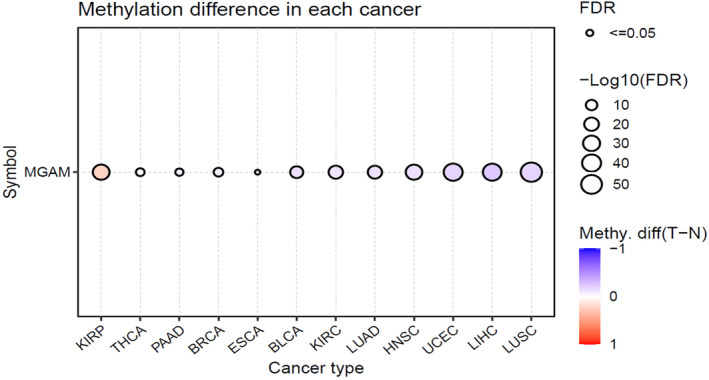
Abnormal *MGAM* methylation patterns were observed in several types of cancers.

Abnormal *MGAM* methylation patterns were observed in several types of cancers, including KIRP, THCA, PAAD, BRCA, ESCA, BLCA, KIRC, LUAD, HNSC, UCEC, LIHC, and LUSC (Figure 5). Furthermore, the relationship between *MGAM* expression and methylation patterns is evident in various cancers such as KIRC, TGCT, KIRP, CHOL, LGG, THYM, STAD, and PAAD, among others (Supporting Information S1: File 5).

### 3.7 MGAM is a potential prognostic biomarker, especially in GI cancers

The findings from multiple analyses in this study highlight the potential prognostic significance of *MGAM* in various cancers, such as BLCA, BRCA, GBM, KIRC, LGG, LUAD, LUSC, OV, SARC, SKCM, THCA, THYM, UCEC, and, interestingly, in most of the GI cancers such as STAD, COAD, ESCA, LIHC, and PAAD (Figure 6). while *MGAM2* was identified as a potential prognostic biomarker for cancers, including ACC, BLCA, BRCA, DLBC, ESCA, KIRC, KIRP, LGG, OV, SKCM, THCA, and UCEC (Supporting Information S1: File 6).

FIGURE 6(A) Pan‐cancer survival analysis of *MGAM* utilizing the cancer genome atlas datasets and Survival package in R. (B) Receiver operating characteristic curve analysis of *MGAM* across different cancers using CombiROC in R.

**Figure:**
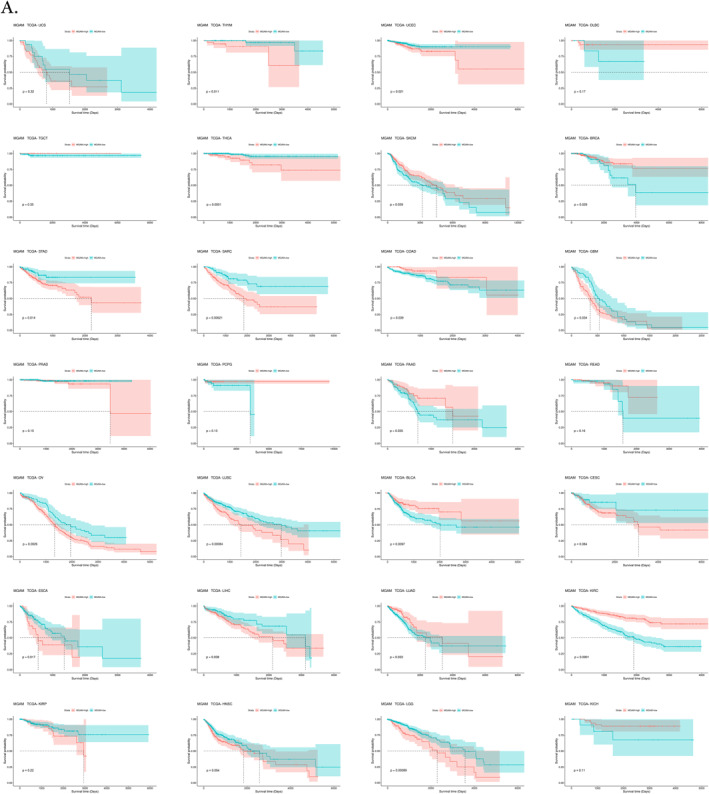

**Figure:**
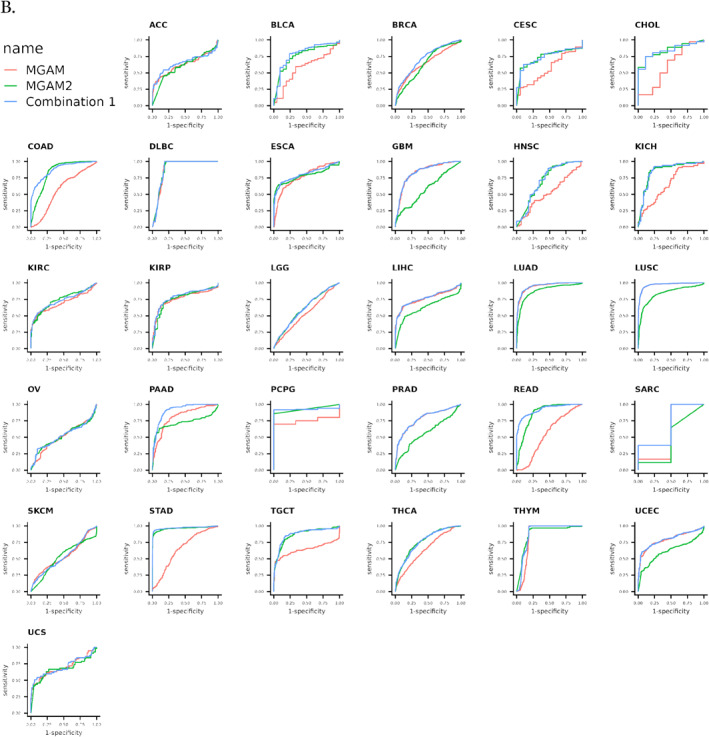

### 3.8 ROC curve analysis showed the diagnostic value of MGAM and MGAM2 in various cancers

ROC curve analysis was conducted to assess the diagnostic efficacy of *MGAM* and *MGAM2* in various cancers. Whereas *MGAM* alone demonstrated diagnostic power exceeding 70% in DLBC, ESCA, GBM, LIHC, KIRP, LUAD, LUSC, PAAD, PCPG, PRAD, THYM, and UCEC cancers, combining *MGAM* with *MGAM2* resulted in increased diagnostic effectiveness. This combination proved particularly potent, offering diagnostic power of over 80% in a broader range of cancers, including CHOL, DLBC, ESCA, GBM, COAD, LUAD, LUSC, PAAD, PCPG, READ, THYM, and STAD (Figure 6B).

### 3.9 Drug‐mRNA interaction analysis suggested MGAM as a potential target for predicting drug sensitivity and resistance

The expression of *MGAM* and its correlation with various drugs were investigated using data from the Cancer Therapeutics Response Portal (CTRP) and GDSC databases. The results revealed a negative correlation between *MGAM* expression and drugs such as PD318088, selumetinib, AICAR, trametinib, tigecycline, (5Z)−7−Oxozeaenol, MLN2480, Dabrafenib, and PLX4720.

**FIGURE 7:**
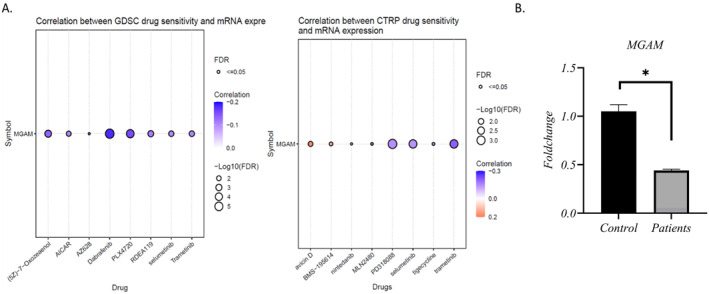
(A) Drug‐mRNA interaction using the gene set cancer analysis database, based on data from genomics of drug sensitivity in cancer and cancer therapeutics response portal. (B) Real‐time PCR analysis of *MGAM* in the CRC patients and normal controls.

Conversely, a positive association was observed with drugs such as avicin D, nintedanib, and BMS‐195614, indicating that increased *MGAM* expression is linked to increased resistance to these drugs (Figure 7A). Moreover, results from the DGIdb database showed that *MGAM* and *MGAM2* were targets of approved antidiabetic drugs such as VOGLIBOSE, ACARBOSE, MIGLITOL, etc., as demonstrated in Table 1.

**TABLE 1:** Drug–target interaction based on DGIdb module (GSCA database).

Gene	Drug	Regulatory approval	Indication	Interaction score
MGAM2	VOGLIBOSE	Approved	Antidiabetic	6.563458904
MGAM2	CELGOSIVIR	Not approved		6.563458904
MGAM	MALTOSE	Approved		2.386712329
MGAM	SALACINOL	Not approved		2.386712329
MGAM	MIGLITOL	Approved	Hypoglycemic agents	4.773424658
MGAM	VOGLIBOSE	Approved	Antidiabetic	4.773424658
MGAM	CELGOSIVIR	Not approved		2.386712329
MGAM	RESVERATROL 4′‐METHYL ETHER	Not approved		9.546849316
MGAM	GLYCOVIR	Not approved		4.773424658
MGAM	LYSOSOMAL ALPHA‐GLUCOSIDASE	Approved		1.591141553
MGAM	BAICALEIN	Not approved		0.367186512
MGAM	DUVOGLUSTAT	Not approved	For the treatment of pompe disease	1.193356164
MGAM	ACARBOSE	Approved	Antidiabetic	3.182283105

### 3.10 Real‐time PCR validated the low expression of MGAM in CRC patients

Our primary objective was to investigate *MGAM* as a direct target of alpha‐glucosidase inhibitor drugs in CRC. Furthermore, factors such as the prognostic value of this gene and its diagnostic potential in combination with *MGAM2* in CRC, along with the association of gene mutations with CRC, suggested that this gene could serve as a potential biomarker for this cancer. Consequently, at this stage, we validated the gene expression in CRC patients using qRT‐PCR. The results showed downregulation of *MGAM* in CRC patients versus controls (Figure 7B).

### 3.11 WES analysis revealed a novel mutation of MGAM

First, we conducted a comprehensive analysis of the SNV file from the COAD‐TCGA dataset to identify pathogenic variants. The results revealed several missense mutations with predicted deleterious effects, such as rs782114142, rs1267900787, and rs1554457449, among others, as presented in Table 2.

**TABLE 2:** Pathogenic variants of MGAM across the COAD‐TCGA dataset.

Hugo_Symbol	Variant_Classification	dbSNP_RS	HGVSc	HGVSp	HGVSp_Short	Existing_variation	SIFT	PolyPhen	Impact	COSMIC
MGAM	Missense_Mutation	rs782114142	c.1265A > G	p.Asp422Gly	p.D422G	rs782114142; COSV72075101	deleterious (0)	probably_damaging (0.998)	MODERATE	COSM269579; COSM269580
MGAM	Missense_Mutation	rs1267900787	c.2351C > T	p.Ala784Val	p.A784V	rs1267900787; COSV101527578	deleterious (0)	probably_damaging (0.952)	MODERATE	NA
MGAM	Missense_Mutation	NA	c.1751A > T	p.Asn584Ile	p.N584I	COSV72074775	deleterious (0)	probably_damaging (0.965)	MODERATE	COSM1448697; COSM1448698; COSM1448699
MGAM	Missense_Mutation	NA	c.4513G > T	p.Gly1505Trp	p.G1505W	COSV72074484	deleterious (0)	probably_damaging (1)	MODERATE	COSM1448740; COSM1448741; COSM1448742
MGAM	Missense_Mutation	rs372476876	c.1847C > T	p.Ala616Val	p.A616V	rs372476876; COSV72073881	tolerated (0.1)	benign (0.294)	MODERATE	COSM1448700; COSM304505; COSM304506
MGAM	Missense_Mutation	rs1554457449	c.344G > A	p.Arg115His	p.R115H	rs1554457449; COSV71885052	deleterious (0.02)	probably_damaging (0.965)	MODERATE	COSM294562; COSM294563; COSM294564
MGAM	Missense_Mutation	rs377213841	c.3748C > T	p.Arg1250Cys	p.R1250C	rs377213841	deleterious (0.01)	probably_damaging (1)	MODERATE	NA
MGAM	Missense_Mutation	rs377213841	c.3748C > T	p.Arg1250Cys	p.R1250C	rs377213841	deleterious (0.01)	probably_damaging (1)	MODERATE	NA
MGAM	Missense_Mutation	NA	c.3725G > T	p.Trp1242Leu	p.W1242L	COSV72074456	deleterious (0.01)	probably_damaging (1)	MODERATE	COSM1448728; COSM1448729; COSM1448730
MGAM	Missense_Mutation	rs778528944	c.2501G > A	p.Arg834Gln	p.R834Q	rs778528944; COSV72073567	deleterious (0)	probably_damaging (1)	MODERATE	COSM599595; COSM599596; COSM599597
MGAM	Missense_Mutation	rs1431849178	c.3287T > G	p.Ile1096Ser	p.I1096S	rs1431849178; COSV101527722	deleterious (0)	probably_damaging (0.968)	MODERATE	NA
MGAM	Missense_Mutation	NA	c.2161C > T	p.Pro721Ser	p.P721S	COSV72074615	deleterious (0)	possibly_damaging (0.903)	MODERATE	COSM1448701; COSM1448702; COSM1448703
MGAM	Missense_Mutation	rs267601326	c.4708C > T	p.Arg1570Cys	p.R1570C	rs267601326; COSV72074844	deleterious (0)	probably_damaging (1)	MODERATE	COSM3634777; COSM3634778; COSM3634779
MGAM	Missense_Mutation	NA	c.3726G > T	p.Trp1242Cys	p.W1242C	COSV72075636	deleterious (0)	probably_damaging (1)	MODERATE	NA
MGAM	Missense_Mutation	NA	c.3368C > A	p.Ser1123Tyr	p.S1123Y	COSV72073742; COSV72074764	deleterious (0)	probably_damaging (0.999)	MODERATE	COSM1448719; COSM1448720; COSM1448721

Next, we performed WES to determine whether any of the identified missense mutations were present in our patient cohort. Interestingly, none of those mutations were detected; however, all 15 individuals consistently harbored a specific variant in the *MGAM* gene, namely rs2960746. Although this variant is currently classified as a variant of uncertain significance (VUS) in the Varsome database, its potential pathogenicity is supported by deleterious predictions from both the SIFT and SIFT4G algorithms. Ongoing functional studies and routine follow‐up investigations are warranted to better clarify the clinical relevance of this variant.

## 4 DISCUSSION

In clinical oncology, where the search for effective treatments against the complex and varied nature of cancer continues, drug repurposing has emerged as a pivotal strategy. This approach leverages existing medications approved for other indications, offering a faster, less costly, and potentially safer alternative to traditional drug development processes. The core objective of drug repurposing is to identify novel therapeutic applications for known compounds, thereby accelerating the delivery of personalized treatments tailored to individual patient needs.^34^

The integration of advanced technologies, such as machine learning and omics, plays a crucial role in this endeavor.^35^, ^36^ These tools enable the prediction of disease–drug pairs through a combination of phenotypic studies, mechanistic investigations, chemical genetics, and omics assays. This multidisciplinary approach is particularly critical for addressing the challenges posed by cancer heterogeneity, recurrence, and metastasis, ensuring rapid and personalized interventions for patients. Omics technologies, encompassing genomics, proteomics, metabolomics, and others, offer unparalleled insights into the molecular mechanisms underlying diseases such as cancer.^37^ By analyzing these “omic” layers, researchers can uncover potential targets for drug repurposing, identifying pathways and molecules that, when modulated, could lead to therapeutic benefits. This omics‐driven approach facilitates the identification of off‐target effects and polypharmacology, where a single compound interacts with multiple targets simultaneously. Such findings are instrumental in refining phenotyping strategies and tailoring treatments to the unique characteristics of each patient's tumor.^38^, ^39^, ^40^

Cancer is increasingly recognized as a metabolic disease, highlighting the importance of metabolic pathways in its development and progression.^41^ Drugs originally developed for metabolic disorders, such as type 2 diabetes mellitus (T2DM), are being repurposed for cancer therapeutics because of shared risk factors and pathophysiological mechanisms. For instance, antidiabetic drugs such as metformin have demonstrated a reduced risk of overall cancer incidence and mortality across various types of cancer.^42^, ^43^, ^44^ Similarly, other antidiabetic agents, such as glyburide and acarbose, showed promise in preclinical models by modulating inflammatory responses and enhancing immune surveillance against cancer.^42^ Studies have shown that acarbose can inhibit tumor growth and enhance immune responses in colon cancer, suggesting a synergistic effect when combined with immunotherapeutic approaches.^45^

The metabolic landscape of the tumor microenvironment often leads to abnormal glycolysis and the subsequent accumulation of lactic acid within tumor cells. This phenomenon can suppress the response of CD8+ T cells, thereby posing a significant challenge in cancer treatment. This underscores the importance of exploring metabolic pathways as targets for repurposing therapy in colorectal cancer, aiming to develop more effective and personalized treatment strategies.^46^, ^47^, ^48^ α‐Glucosidase inhibitors, such as acarbose, voglibose, and miglitol, are utilized for their efficacy in managing type 2 diabetes. These drugs act by targeting the maltase–glucoamylase enzyme (MGAM) and play a crucial role in the final stages of starch digestion.^16^ Therefore, to gain a clearer understanding of the role and potential significance of *MGAM* in the molecular mechanisms underlying cancer, with the ultimate goal of identifying a target for drug repurposing strategies in cancer treatment, we conducted a comprehensive pan‐cancer analysis of *MGAM*. This pioneering analysis encompassed an examination of gene expression levels, mutations, methylation patterns, immune cell infiltration, and the association of drugs with this gene. Following this, we delved into the implicated gene pathways and protein–protein interactions, assessing *MGAM*'s and its paralog *MGAM2's* potential as a prognostic and diagnostic marker in cancer.

In the present study, the comprehensive analysis of pooled data from multiple resources, including TCGA, GTEX datasets, Firebrowse, and GSCA databases, has unveiled substantial variations in the expression levels of the *MGAM* gene across a wide spectrum of cancer types. These types encompassed PAAD, ESCA, KIRC, GBM, BLCA, DLBC, LUAD, LUSC, PRAD, THYM, BRCA, SARC, and STAD. It is particularly noteworthy that, based on the results from online databases, *MGAM* demonstrated a diminished expression trend in gastrointestinal (GI) cancers, which include COAD, READ, CHOL, ESCA, LIHC, and STAD.

To enrich our comprehension of *MGAM's* function, we broadened our research to encompass its lesser‐known variant, *MGAM2*, scrutinizing its differential expression across various forms of cancer. Our results suggested an elevation in *MGAM2* expression in conditions such as CESC, CHOL, COAD, ESCA, KIRC, LIHC, PCPG, READ, SKCM, STAD, and downregulation in DLBC, HNSC, KICH, LUAD, TGCT, and THYM. Interestingly, the expression pattern of *MGAM* and *MGAM2* seems to be similar in cancers such as DLBC, KIRC, LUAD, and THYM, whereas it seems to have a converse effect in GI cancers such as COAD, READ, CHOL, ESCA, LIHC, and STAD.

The STRING database mapped the PPI network of the *MGAM* gene, revealing its involvement in various biological processes and MF. Gene ontology analysis revealed that *MGAM* is primarily associated with carbohydrate catabolism and cellular polysaccharide breakdown within metabolic pathways. *MGAM* exhibited specific enzymatic activities, including glucan 1,4‐alpha‐glucosidase, alpha‐glucosidase, and alpha‐1,4‐glucosidase, in collaboration with *MGAM2*. Furthermore, *MGAM* interacted with several CC, such as ficolin‐1‐rich granules, secretory granule lumens, and extracellular exosomes, highlighting its multifaceted role within cells. The GSCA database also predicted a significant association between *MGAM*, *MGAM2*, and various cancer‐related pathways. In several cancers, including BRCA, STAD, ESCA, LIHC, LUSC, HNSC, TGCT, KIRC, and KIRP, *MGAM* influenced pathways such as apoptosis, cell cycle, DNA damage, RTK, PI3KAKT, TSCmTOR, EMT, hormone AR, hormone ER, and RASMAPK. Interestingly, *MGAM's* role varied across these cancers, exhibiting both stimulatory and inhibitory effects on these pathways. This suggests that *MGAM* may promote cancer progression through diverse mechanisms, depending on the specific cancer type and its genetic and molecular context which needs further clarification.

Analysis of *MGAM* mutations showed a high mutation rate across various cancers, including SKCM, UCEC, LUSC, LUAD, HNSC, COAD, BRCA, and STAD. Significant correlations were identified between *MGAM*'s SNVs, OS, and PFS in PRAD, and between *MGAM*'s CNVs and survival metrics in UCEC. Additionally, *MGAM* expression correlated strongly with CNVs in OV, HNSC, THCA, SKCM, LUAD, BRCA, and LGG cancers.

Tumor development and occurrence are intricately linked to the infiltration of immune cells. Tumor immune cell infiltration denotes the migration of immune cells from the bloodstream into the tumor tissue to exert their effects. This phenomenon is closely associated with clinical outcomes and has the potential to serve as a target for drug intervention to enhance patient survival.^49^

Our investigation into the role of *MGAM* and *MGAM2* genes in various cancers has unveiled intriguing insights into their correlation with immune cell infiltration and their potential as diagnostic and prognostic markers. Specifically, we found a positive correlation with neutrophil and monocyte infiltration, yet a negative correlation with CD4+, CD8+ T cells, and DCs across a broad spectrum of cancers, including KIRC, THCA, THYM, BRCA, LAML, PRAD, COAD, HNSC, LIHC, LUAD, STAD, and LGG. Notably, the strongest correlations were observed in LIHC, LGG, STAD, LUAD, SKCM, LAML, and HNSC, suggesting a significant impact of these genes on immune cell dynamics within tumor microenvironments.

Moreover, abnormal methylation patterns of *MGAM* were detected in numerous cancer types, underscoring the gene's involvement in cancer biology beyond its transcriptional regulation. The relationship between *MGAM* expression and methylation further complicates the gene's role in cancer progression, highlighting the need for comprehensive epigenetic analyses.

The prognostic implications of *MGAM* are underscored by our findings, particularly in GI cancers. *MGAM* showed prognostic capability in BLCA, BRCA, GBM, KIRC, LGG, LUAD, LUSC, OV, SARC, SKCM, THCA, THYM, UCEC, and, interestingly, in most of the GI cancers such as STAD, COAD, ESCA, LIHC, and PAAD, while *MGAM2* was identified as a potential prognostic biomarker for cancers, including ACC, BLCA, BRCA, DLBC, ESCA, KIRC, KIRP, LGG, OV, SKCM, THCA, and UCEC. It seemed that *MGAM* emerged as a promising prognostic biomarker specifically for GI cancers. This finding underscores the critical role of *MGAM* in predicting outcomes and guiding treatment decisions in GI malignancies.

The diagnostic accuracy of combining *MGAM* and *MGAM2* gene analysis exceeds 80% across multiple cancer types, including DLBC, ESCA, GBM, COAD, LUAD, LUSC, PAAD, PCPG, READ, THYM, and STAD. This high accuracy underscores the potential of these genes as biomarkers for cancer diagnosis.

Investigations into *MGAM*'s correlation with drug responses revealed complex associations, with increased *MGAM* expression correlating negatively with certain drugs' efficacy and positively with others. These findings suggest a nuanced interplay between *MGAM* expression and drug sensitivity/resistance profiles, which could inform personalized treatment strategies. Furthermore, *MGAM* and *MGAM2* were identified as targets of approved antidiabetic drugs, adding another layer to their multifaceted roles in cancer biology and therapeutics. This discovery opens avenues for exploring the therapeutic potential of targeting *MGAM* in conjunction with existing treatments.

Our findings suggested that *MGAM* could serve as a promising biomarker for CRC. To validate these, we conducted gene expression analysis in CRC patients using qRT‐PCR, which confirmed low expression in CRC patients compared to controls.

The clinical significance of NGS‐based methods is steadily growing. WES stands out as a burgeoning and dependable technology for pinpointing mutational patterns in cancer.^50^ In this research, WES analysis revealed a distinct variant of the *MGAM* gene, specifically rs2960746, consistently present in all 15 CRC patients. This variant was deemed pathogenic through predictions from the SIFT and SIFT4G databases. Conducting functional investigations in this area and regularly updating predictive databases can enhance our understanding of the mutation's phenotypic impact in the future. Furthermore, we assessed deleterious mutations in this gene based on SNV data from COAD‐TCGA.

Several studies were conducted on the importance of *MGAM* in cancer: The significance of the *MGAM* gene in various types of cancer has been extensively explored through network pharmacology and molecular docking technologies. Studies have highlighted *MGAM* as a critical target for traditional Chinese herbal formulas such as Mahuang Fuzi Xixin Decoction (MFXD) in treating lung cancer, particularly in LUAD, where it is a key mutated gene^51^, ^52^ In non‐small cell lung cancer (NSCLC), nearly 18% of patients with specific EGFR mutations also presented *MGAM*–*BRAF* fusions, underscoring the gene's importance in this context.^53^ Recent advancements in examining driver mutations in cancer patients have provided valuable insights for personalized targeted immunotherapy. Mutations in *MGAM* have been linked to enhanced response rates, increased PD‐L1 expression, and higher TMB levels in NSCLC patients, suggesting a potential role in modulating tumor‐infiltrating immune cells.^54^ The distinct N‐glycosylation pattern of *MGAM* serves as a biomarker for bladder cancer progression.^55^ However, its reduced expression in early intestinal cancer suggests its potential as a serum biomarker for early detection.^56^ In a study focusing on three urogenital cancers and benign prostatic hyperplasia (BPH), researchers discovered a strong association between *MGAM* glycoproteins and aggressive prostate cancer. These glycoproteins were found to be uniquely expressed in urine samples from individuals with prostate cancer.^57^ In another study, He et al. proposed a prognostic model comprising seven coding genes, among which *MGAM* was strongly linked to biochemical recurrence after radical prostatectomy in prostate cancer.^58^ *MGAM* exhibited higher expression levels in castration‐resistant prostate cancer metastatic tumors when compared to primary tumors and normal tissue,^59^ which is consistence with our study.

SNV mutations in the *MGAM* gene are more prominently expressed in the glycolytic subtype of cutaneous melanoma (SKCM), which is associated with the poorest prognosis compared to other subtypes.^60^ In gastric cancers, *MGAM* is among the most aberrantly expressed mRNAs, with decreased expression observed in intestinal‐type gastric cancer.^61^ The genome‐wide analysis conducted using complementary DNA microarray has revealed that *MGAM* is among the genes exhibiting decreased expression in intestinal‐type gastric cancer, suggesting its involvement in carcinogenesis.^62^ Genomic comparative hybridization analysis using cDNA microarray was conducted on 30 patients with gastric cancer. The analysis revealed an increased proliferation of regions on chromosome 7, including the *MGAM* gene, which is a target for drug development.^24^ Vincent‐Chong et al.'s study identified copy number alterations (CNA) potentially linked to cancer in oral squamous cell carcinoma (OSCC) through high‐resolution array comparative genomic hybridization (aCGH). Genomic analyses have identified *MGAM* as a target for drug development because of its amplified expression in OSCC. Furthermore, the study demonstrated a 6.6‐fold increase in *MGAM* expression in OSCC.^63^ Shi et al. have reported that *MGAM* exhibits a distinct expression pattern in breast cancer among Caucasian and Asian Americans.^64^ In research conducted by Shutan Xu and colleagues, it was revealed that the expression of *MGAM2* is predominantly found in BLBCs. Interestingly, this expression correlates with improved survival rates among breast cancer patients. In contrast, *MGAM* is more commonly associated with luminal A breast cancers. The identification of *MGAM2* and other ER resident proteins, such as ERV1 homologs (EHVs), has implications for cancer immunotherapy and the treatment of BLBCs. The study further expanded on the distribution of *MGAM2*, demonstrating its presence across various tissues beyond just the breast. These tissues include the blood and the GI tract, indicating a broader role for *MGAM2* in the body. Notably, there was a significant correlation between *MGAM2* expression and gene signatures indicative of immune cell activity, especially neutrophils within the bloodstream. Moreover, the findings underscored the relevance of *MGAM2* expression in GI cancers, suggesting a potential link between *MGAM2* and these types of malignancies.^20^

WES analysis conducted on patients with anal canal squamous cell carcinoma (ACSCC) revealed that *MGAM2* ranks among the top three most commonly mutated genes in this uncommon form of cancer.^65^ *MGAM2* has been linked to cancers associated with the carbohydrate metabolism pathway. Additionally, this gene was connected to patients with hereditary CRC and simultaneous GC.^66^ Recently, a study has identified deleterious variants in the *MGAM* gene that may be linked to monogenic or oligogenic inheritance in cases of IBS. This discovery has positioned *MGAM* as a potential target for personalized treatment in individuals with this syndrome.^67^

Whereas our study focuses on CRC, the widespread involvement of *MGAM* in various cancer types suggests its crucial role in cancer biology. The association of *MGAM* with cancers linked to carbohydrate metabolism pathways underscores the importance of metabolic reprogramming in cancer. This connection highlights the potential benefits of targeting metabolic pathways in cancer therapy. However, the complexity of cancer biology means that no single gene can fully predict treatment outcomes. Future research with functional studies should aim to integrate *MGAM* expression data with other molecular markers to develop more comprehensive predictive models for cancer therapy.

In light of the comprehensive analysis of *MGAM* and *MGAM2* gene expressions within various cancer contexts, it is clear that these genes represented pivotal players in the complex nature of cancer biology. Their association with distinct cancer subtypes, disease progression stages, and immune cell dynamics not only sheds new light on the underlying mechanisms of cancer but also paves the way for the development of innovative diagnostic tools and prognostic indicators. Furthermore, the intriguing link between *MGAM* and alpha‐glucosidase inhibitors opens up exciting avenues for repurposing existing drugs in oncology, thereby offering hope for more effective and less toxic treatments. As our understanding deepens through continuous research and the identification of specific genetic variants like rs2960746, the potential of *MGAM* and *MGAM2* in revolutionizing cancer care becomes even more apparent. However, conducting functional studies and updating databases will comprehensively complete our understanding of these genes' roles in cancer. This commitment will be crucial in leveraging the full potential of *MGAM* and *MGAM2* in advancing personalized medicine and ultimately enhancing patient outcomes.

**In conclusion**, our investigation provides robust evidence supporting the critical function of the *MGAM* and *MGAM2* genes in cancer biology. This discovery opens up promising directions for further exploration into their utility in prognostication, diagnosis, and treatment strategies. To the best of our knowledge, this study marks the inaugural comprehensive examination of *MGAM* across a spectrum of cancer types. The implications of our findings highlight the potential of *MGAM* as a significant biomarker for assessing cancer prognosis. Furthermore, they offer valuable insights into the possible pathways through which *MGAM* might influence tumor development.

## AUTHOR CONTRIBUTIONS

Conceptualization: Hanieh Azari; Methodology: Farzaneh Alizadeh, Rawaa Chasib Mezher, Hamid Fiuji and Hanieh Azari; Software: Reza Khayami and Hanieh Azari; Validation: Ibrahim Saeed Gataa; Formal analysis: Hamid Fiuji; Investigation: Hanieh Azari, Farzaneh Alizadeh, Majid Rajabian; Resources: Ladan Goshayeshi; Data curation: Reza Khayami; Writing—original draft preparation: Rawaa Chasib Mezher and Hanieh Azari; Writing—review and editing: Majid Rajabian; Visualization: Reza Khayami and Hanieh Azari; Supervisions: Seyed Mahdi Hassanian and Amir Avan; Project administration: Amir Avan: Funding acquisition: Amir Avan. All authors have read and agreed to the published version of the manuscript.

## CONFLICT OF INTEREST STATEMENT

The authors declare no conflicts of interest.

## ETHICS STATEMENT

Ethical approval for this study was obtained from the Mashhad University of Medical Sciences Review Board (IR.MUMS.MEDICAL.REC.1402.389).

## Supporting information

Supporting Information S1

## Data Availability

The data that support the findings of this study are available on request from the corresponding author upon reasonable request.
