# Supplementary figures and images for: Pan‐cancer analysis and oncogenic implications of MGAM and MGAM2: Toward precision oncology and drug repurposing in colorectal cancer
Source: J Cell Commun Signal. 2025 Aug 27;19(3):e70042. doi: 10.1002/ccs3.70042 (PMC12381534; doi:10.1002/ccs3.70042)

## Slide 1
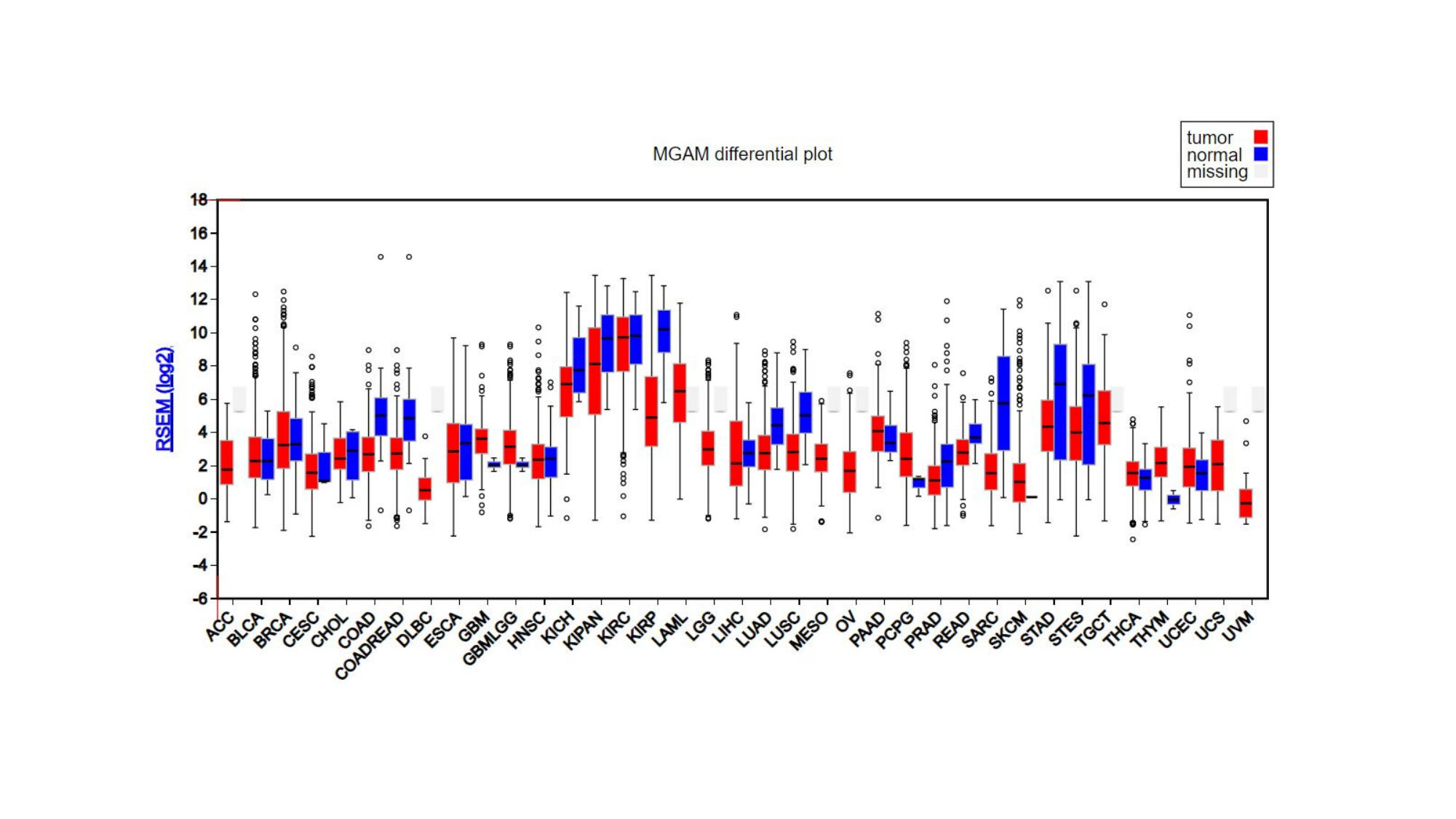

## Slide 2
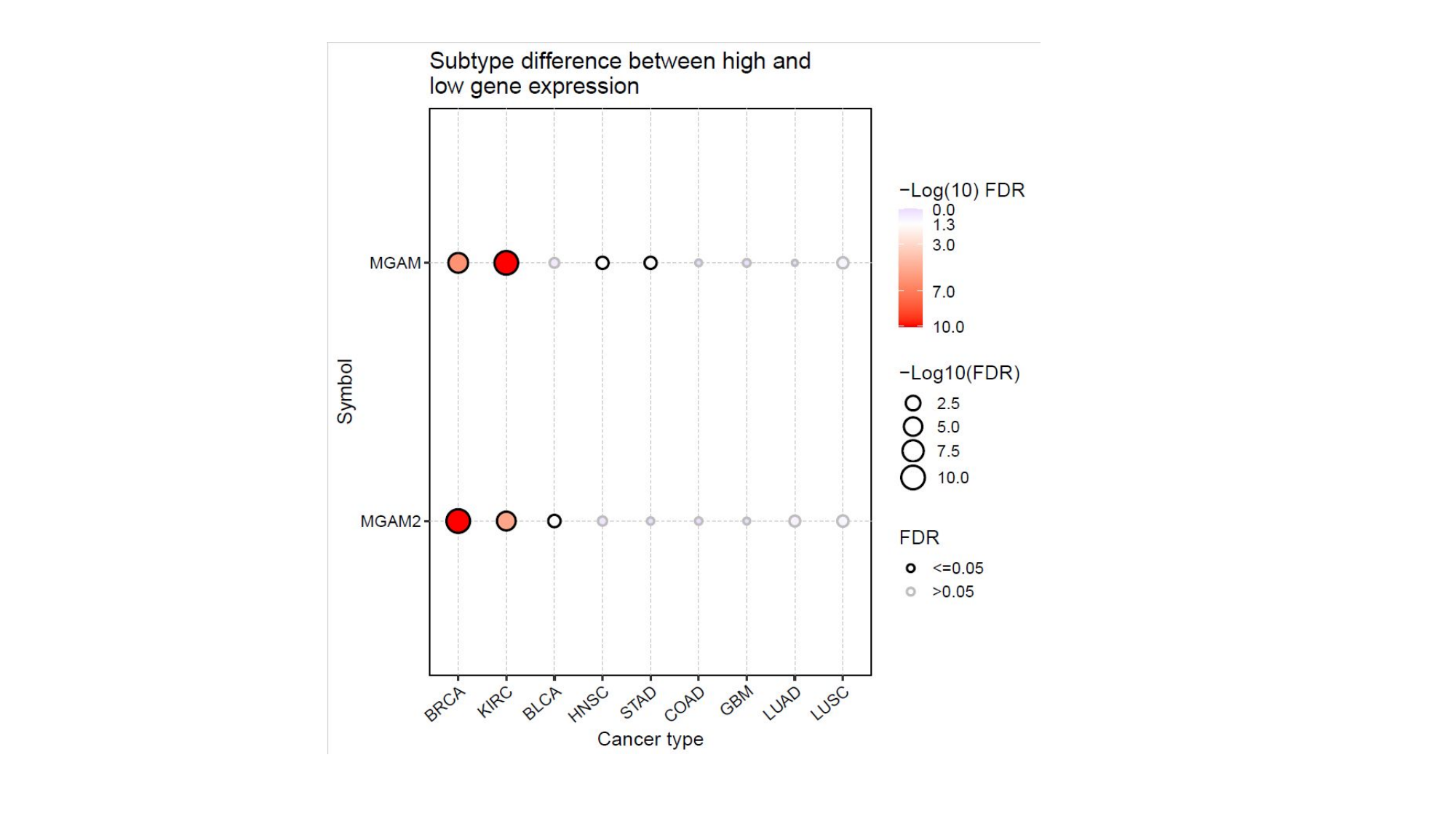

## Slide 3
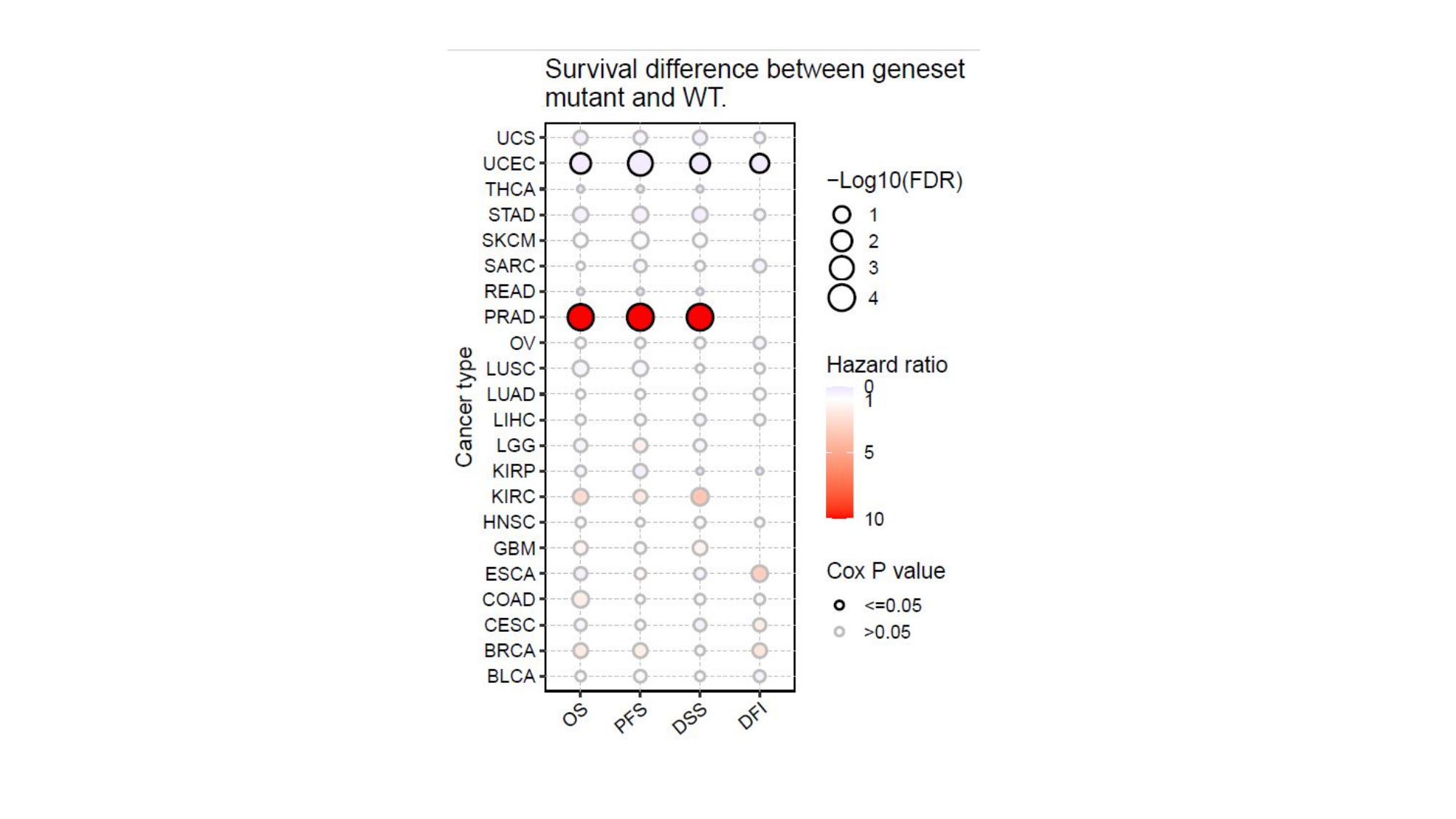

## Slide 4
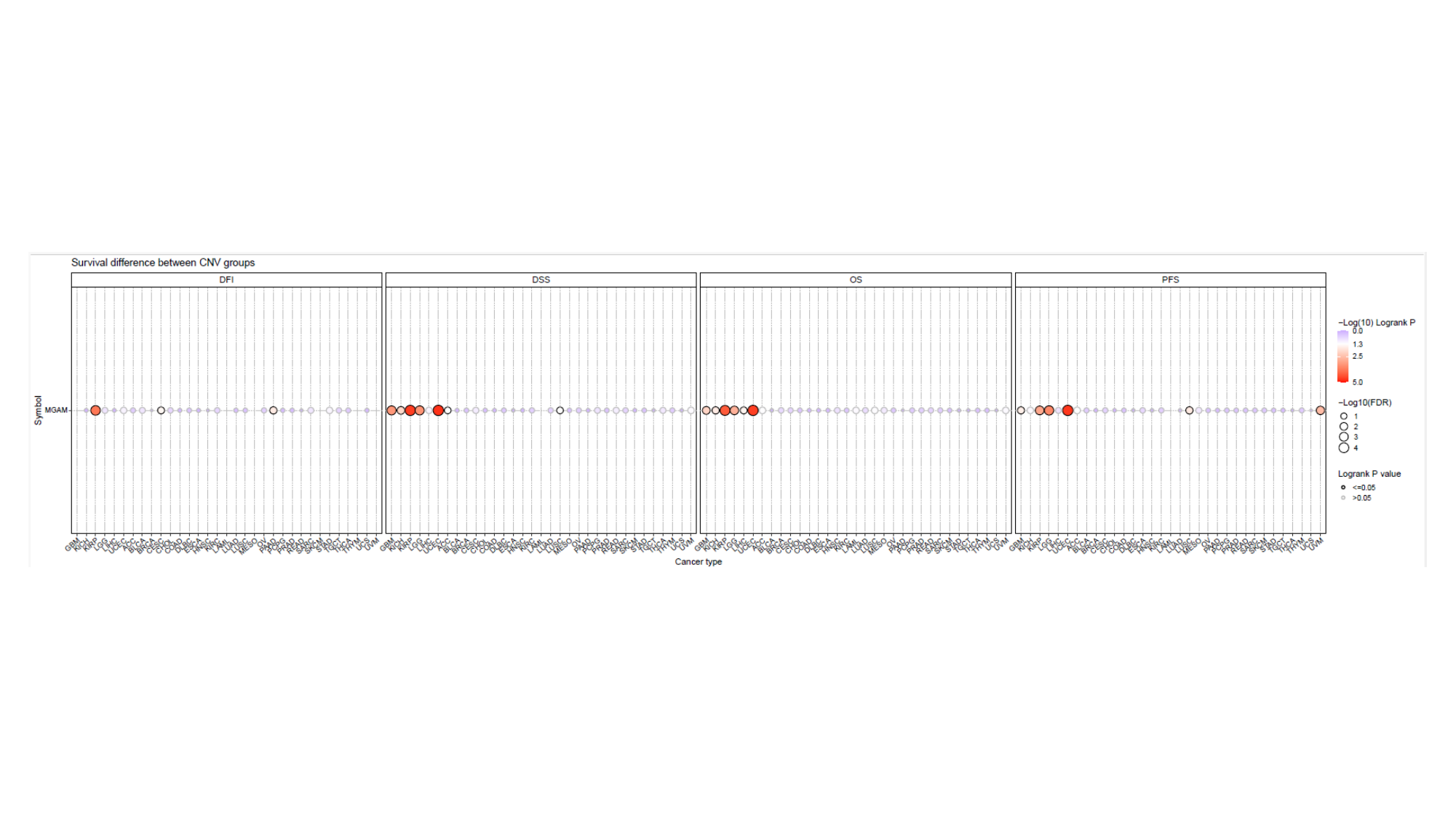

## Slide 5
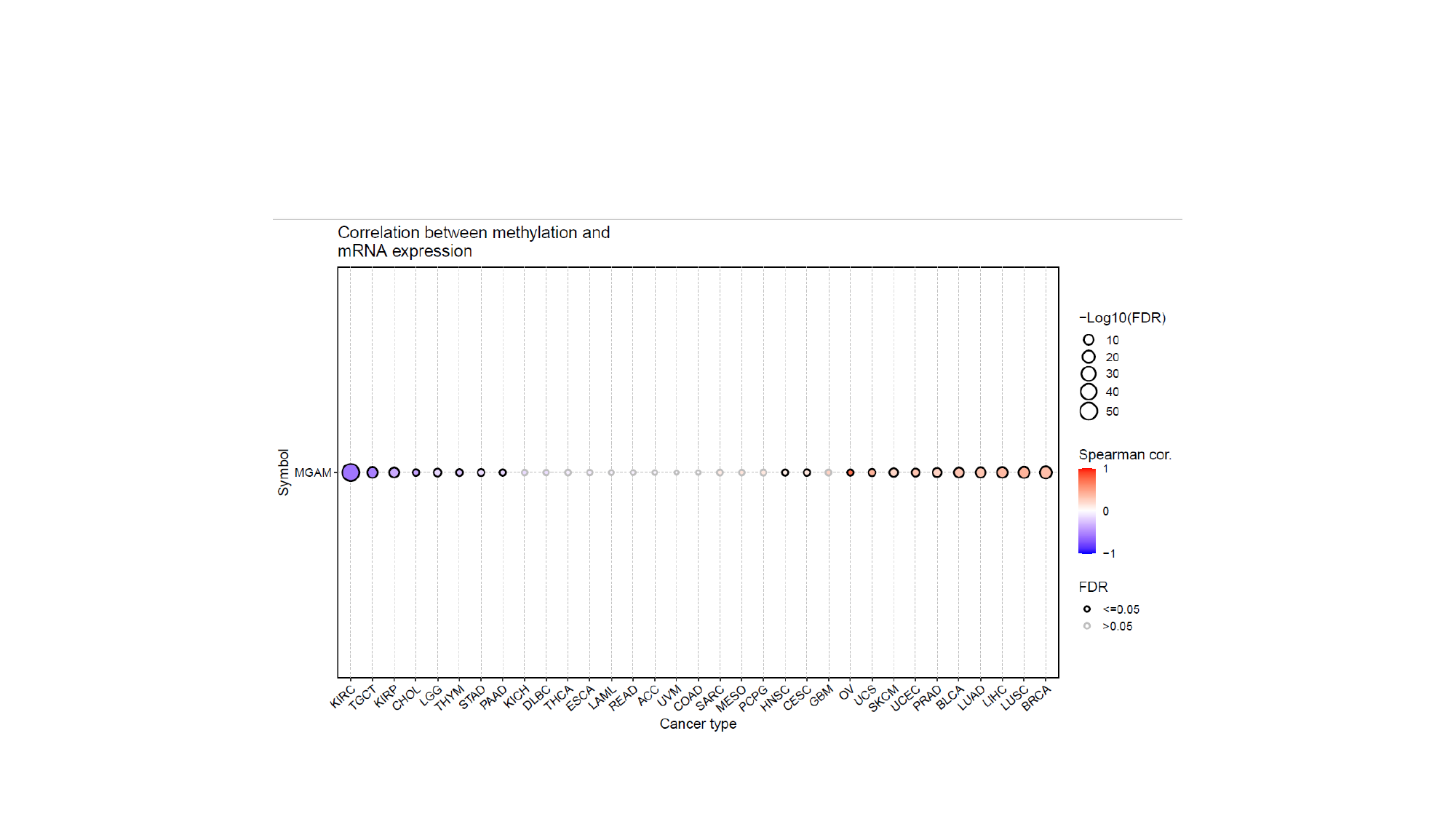

## Slide 6
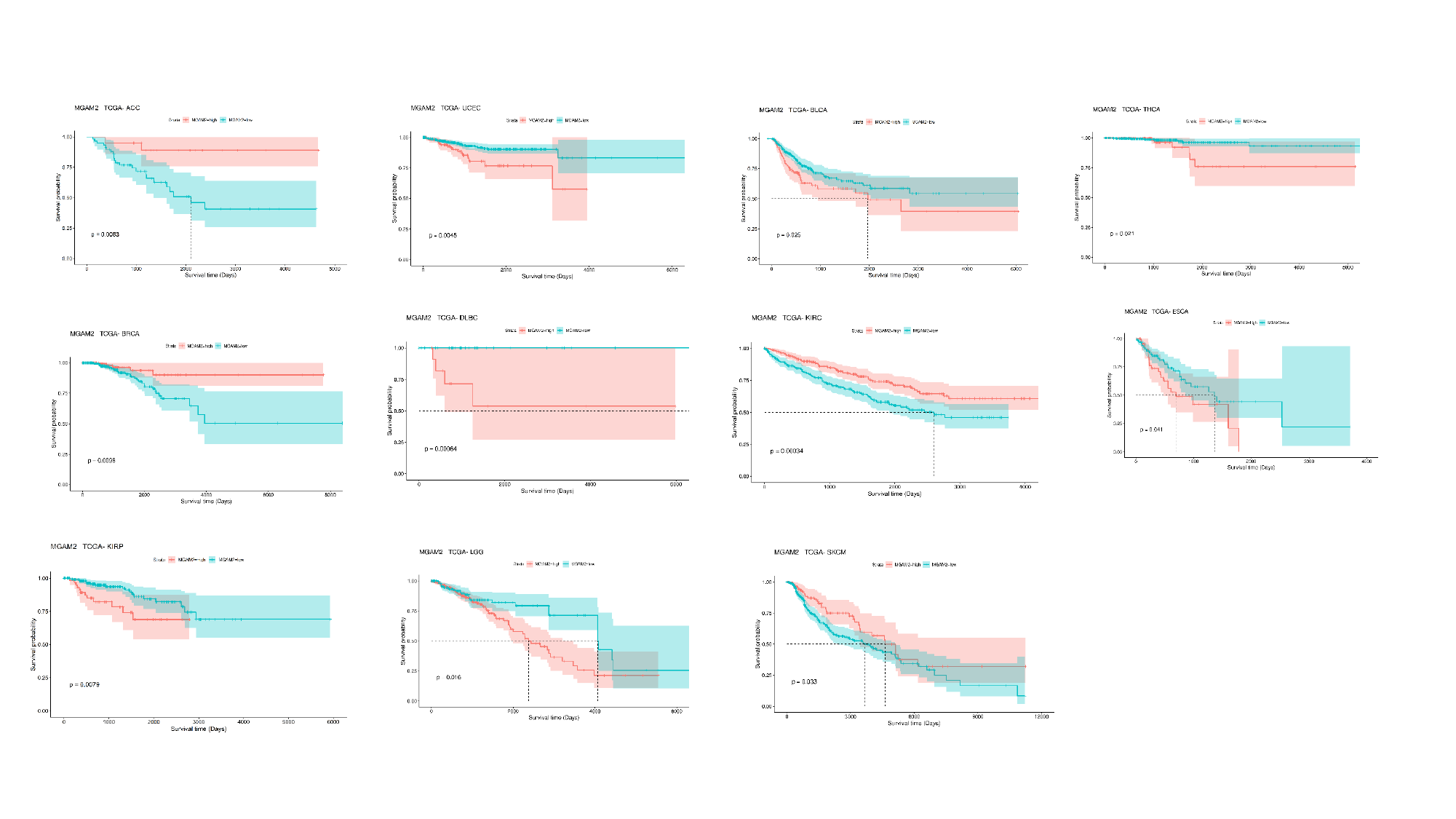

## Slide 7
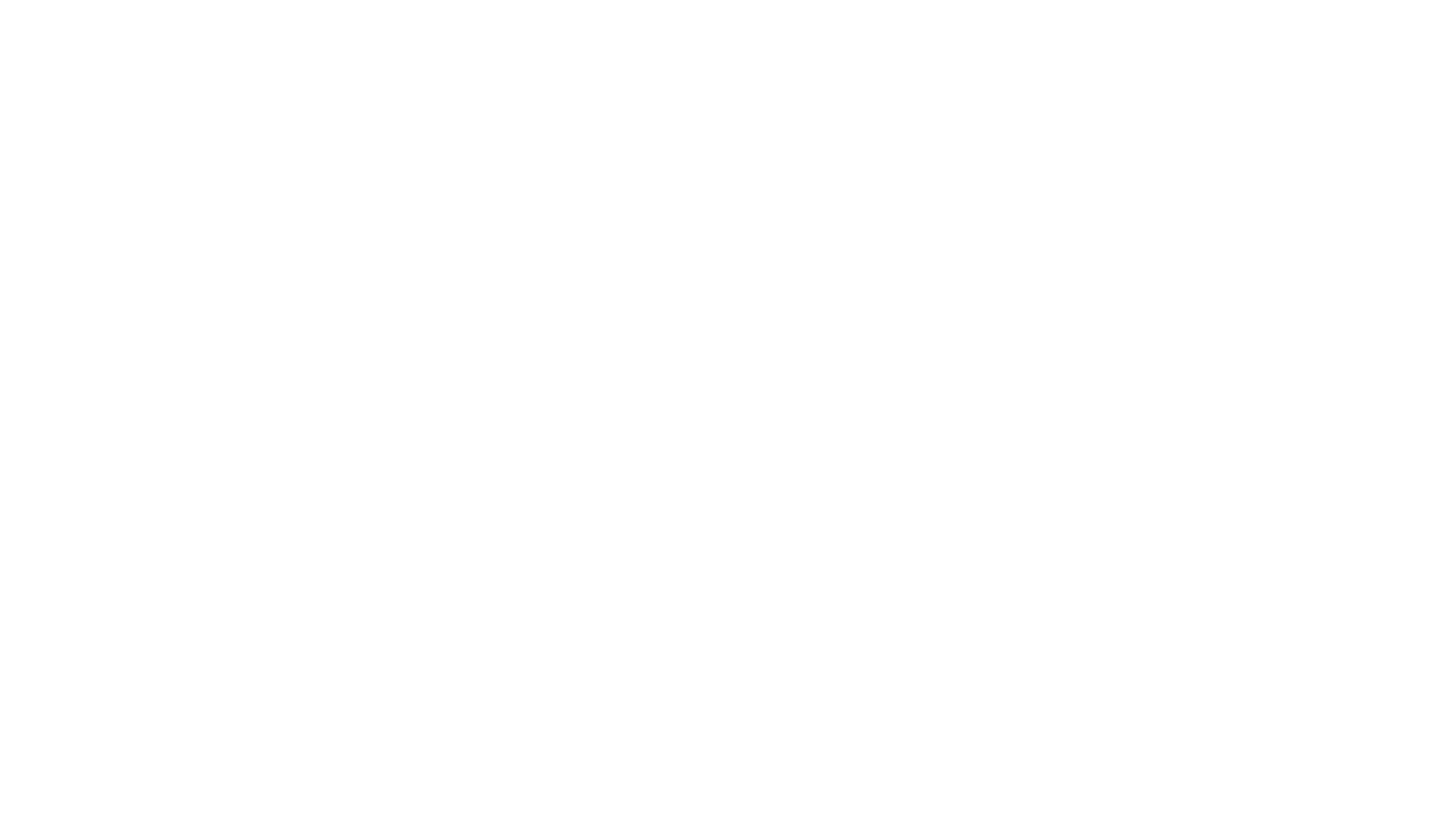

#

Supplement: Supplementary file 1 — Supporting Information S1 [file CCS3-19-e70042-s001.pptx]
